# Disturbed functional brain networks and neurocognitive function in low-grade glioma patients: a graph theoretical analysis of resting-state MEG

**DOI:** 10.1186/1753-4631-3-9

**Published:** 2009-08-23

**Authors:** Ingeborg Bosma, Jaap C Reijneveld, Martin Klein, Linda Douw, Bob W van Dijk, Jan J Heimans, Cornelis J Stam

**Affiliations:** 1 Department of Neurology, VU University Medical Center, Amsterdam, the Netherlands; 2 Department of Neurology, Academic Medical Center, Amsterdam, the Netherlands; 3 Department of Medical Psychology/MEG, VU University Medical Center, Amsterdam, the Netherlands; 4 Department of Clinical Neurophysiology/MEG, VU University Medical Center, Amsterdam, the Netherlands

## Abstract

**Background:**

To understand neurophysiological mechanisms underlying cognitive dysfunction in low-grade glioma (LGG) patients by evaluating the spatial structure of 'resting-state' brain networks with graph theory.

**Methods:**

Standardized tests measuring 6 neurocognitive domains were administered in 17 LGG patients and 17 healthy controls. Magnetoencephalography (MEG) recordings were conducted during eyes-closed 'resting state'. The phase lag index (PLI) was computed in seven frequency bands to assess functional connectivity between brain areas. Spatial patterns were characterized with graph theoretical measures such as clustering coefficient (local connectivity), path length (global integration), network small world-ness (ratio of clustering coefficient/path length) and degree correlation (the extent to which connected nodes have similar degrees).

**Results:**

Compared to healthy controls, patients performed poorer on psychomotor functioning, attention, information processing, and working memory. Patients displayed higher short- and long-distance synchronization and clustering coefficient in the theta band, whereas a lower clustering coefficient and small world-ness were observed in the beta band. A lower degree correlation was found in the upper gamma band. LGG patients with higher clustering coefficient, longer path length, and lower degree correlations in delta and lower alpha band were characterized by poorer neurocognitive performance.

**Conclusion:**

LGG patients display higher short- and long-distance synchronization within the theta band. Network analysis revealed changes (in particularly the theta, beta, and upper gamma band) suggesting disturbed network architecture. Moreover, correlations between network characteristics and neurocognitive performance were found, Widespread changes in the strength and spatial organization of brain networks may be responsible for cognitive dysfunction in glioma patients.

## Background

Gliomas are primary brain tumors originating from glial tissue. Twenty to twenty-five percent of these tumors are low grade gliomas (LGG) [1]. During the disease course, most LGG patients are confronted with a loss of neurocognitive functioning, which tends to have a global character and cannot be explained by tumor localization alone [2,3]. Higher neurocognitive functioning depends on both focal processing in different brain regions and global integration of neuronal activity [4].

Brain activity can be registered with magnetoencephalography (MEG), which records magnetic fields related to intracellular neuronal currents. Statistical correlations of the activity recorded over the different brain regions are thought to reflect functional interactions between brain regions. This concept is referred to as 'functional connectivity' [5,6]. Functional connectivity has been studied in brain tumor patients with different methods [7-10]. In a resting state, significant differences compared with healthy controls were found regarding synchronization in different frequency bands [7-9], and associations of functional connectivity with neurocognitive functioning were reported [9]. In these studies, the synchronization likelihood (SL) was used as a measure of statistical interdependencies between the MEG time series [11], based on the concept of general synchronization [12]. Another study used the phase lag index (PLI) to evaluate changes in functional connectivity in brain tumor patients before and after surgery [10]. A significant decrease in theta band functional connectivity was found after surgery, which was hypothesized to be a result of a normalization due to the resection of the lesion.

The PLI is a novel method that can be used to detect synchronous neural activity of the brain in EEG and MEG recordings by detecting nonzero phase difference coupling. This method has been shown to be less sensitive to volume conduction than other measures of functional connectivity [13].

Up till now, it remains unclear whether changes in the mean level of coupling are also associated with changes in the global organization of functional networks. A method of characterizing these complex networks is the use of the graph theory, a field of mathematics that is particularly useful to describe the organization. A graph is a representation of a network which is reduced to vertices (nodes) and their edges (connections) and can be described by several measures such as the clustering coefficient (C) and the path length (L). The clustering coefficient (C) is a measure of the local structure, indicating the proportion of neighboring vertices that are interconnected. The path length (L) describes the global integration by measuring the mean number of steps to go from one vertex to another vertex. By computing these two parameters, networks can be classified as regular (in which there is a high local interconnectedness characterized by a high C, and a long path length characterized by a high L) or random (with a low local clustering characterized by a low C, and a short path length characterized by a low L). Watts and Strogatz introduced a novel, intermediate type of graph characterized by a high local interconnectedness (high C) and a short path length (low L), which they called a "small-world network" [14]. Many networks (social networks, neural network of the C. Elegans and man-made networks such as the architecture of the power grid system in the USA) show small-world features. The specific architecture of these small-world networks is thought to be the optimal topography for many processes depending on network interactions, including information processing in the brain [15,16]. Graph theoretical properties of neural networks have been studied before in healthy subjects [17-21], and in patients with brain pathology such as Alzheimer's disease (AD) [22,23], schizophrenia [24,25] and brain tumors [8]. In the study of Bartolomei, alterations in network architecture were found: brain tumor patients showed a tendency for these networks to have a more random configuration [8]. It was suggested that these alterations might be associated with the global loss of neurocognitive function brain tumor patients are confronted with. This hypothesis could not be tested, since no data of neuropsychological assessments were available. To test this interesting hypothesis we performed a new study with a more homogenous patient population and matched healthy controls and collected both resting state MEG data of both groups as well as their results on neuropsychological assessments.

In the present study, we therefore investigated graph theoretical properties and neurocognitive functioning in LGG patients. We hypothesize that functional connectivity determined by the PLI varies between LGG patients and healthy controls, and secondly we expect to find evidence for a loss of small-world network characteristics in patients which we expect to be correlated with neurocognitive functioning.

## Methods

### Patients and controls

Between April and November 2005, all LGG patients who attended the outpatient clinic of the VU University Medical Center (VUmc) and the Academic Medical Center (AMC), both tertiary referral centers for brain tumor patients in Amsterdam, The Netherlands, were approached for participation in this study. The results of power analysis and functional connectivity (using the SL) of this cohort have been published previously [9,26]. In short, patients were eligible if: (a) they had a suspected or histologically confirmed low-grade glioma (LGG); (b) there was no radiological (confirmed by MR or CT scan) and/or clinical tumor progression in the previous 6 months; (c) they did not use medication possibly interfering with neurocognitive functioning, other than anti-epileptic drugs (AEDs).

The study was approved by the institutional ethical review boards of both participating hospitals. Relatives of the patients were asked to participate as healthy controls. Healthy controls were eligible if they: (a) did not suffer from any neurological disease; (b) did not use any medication possibly influencing neurocognitive functioning. For patients who could not provide a healthy control participant, VU University Medical Center staff members were included.

### Neurocognitive assessment

Participants were asked to complete a neurocognitive assessment (see table 1[27-35]) after MEG recording. Individual patients' test scores were converted to *z*-scores, using the means and standard deviations of the matched healthy controls as a reference. To reduce data, individual *z*-scores on these tests were summarized into six neurocognitive domains (respectively 1) information processing speed, 2) psychomotor function, 3) attention, 4) verbal memory, 5) working memory and 6) executive functioning). Construction of these domains has been reported previously [36], and was based on a Principal Component Analysis using Varimax rotation with Kaiser normalization performed on the *z*-scores of a large group of healthy controls [37]. The domains found are commonly used in neurocognitive practice and research. An overall measure of cognition was also determined by calculating the mean of all test *z*-scores for each participant.

**Table 1:** Description of neuropsychological testing battery

**test**	**Cognitive abilities**
Letter Digit Substitution Test (LDST) [27,28]	Psychomotor function relatively unaffected by intellectual ability

Concept Shifting Test [29,30]	Executive (frontal) function, attention, visual scanning and mental processing speed

Stroop Color Word Test [31,32]	Executive (frontal) function, attention, mental speed and mental control

Visual Verbal Learning Test [33]	Various aspects of verbal learning, organisation and memory

Memory Comparison Test [34]	Selective attention, mental concentration, memory and information processing

Categoric Word Fluency [35]	Frontal dysfunction and flexibility of verbal thought processes

### Magnetoencephalography

Magnetoencephalography (MEG) recordings were obtained using a 151-channel whole-head MEG system (CTF systems; Port Coquitlam, BC, Canada) while participants were seated inside a magnetically shielded room (Vacuumschmelze GmbH, Hanau, Germany). Magnetic fields were recorded during a no-task, eyes-closed resting state. Metal artefacts were avoided as much as possible. A third-order software gradient was used with a recording passband of 0.25 to 125 Hz and a sample frequency of 312.5 Hz.

At the beginning, middle, and end of each recording, the head position relative to the coordinate system of the helmet was recorded by leading small alternating currents through three head position coils attached to the left and right preauricular points and the nasion on the subject's head. Head position changes up to approximately 1.5 cm during a recording condition were accepted.

For this study, 149 of the 151 channels could be used. MEG recordings were converted to ASCII files. From these ASCII files four artefact free epochs of 13 seconds (4,096 samples) were carefully selected by visual analysis (IB).

Magnetic field frequencies ranging from 0.5 to 80 Hz were recorded. The signals were then filtered into seven frequency bands (respectively delta (0.5–4 Hz), theta (4–8 Hz), lower alpha (8–10 Hz), upper alpha (10–13 Hz), beta (13–30 Hz), lower gamma (30–45 Hz), and upper gamma band (55–80 Hz)).

### Functional connectivity

Functional connectivity was assessed with the phase lag index (PLI), calculating the asymmetry of the distribution of (instantaneous) phase differences between two MEG signals. The PLI ranges between 0 and 1, and a PLI of more than 0 indicates phase locking to a certain extent, whereas a PLI of 0 indicates no coupling or coupling with a phase difference centered around 0 ± π radians. It assumes that the presence of a consistent, nonzero phase lag between two time series cannot be explained by volume conduction alone. Thus, finding true interactions instead of volume conduction effects is more likely when using this method [13].

PLI values were calculated between all possible pair-wise combinations of MEG sensors for all frequency bands separately, and the results of four epochs were averaged for each participant. Two types of PLI scores were then calculated: (1) ten (five per hemisphere) short-distance PLIs (PLI scores within 1 MEG region, respectively left and right frontal, parietal, central, occipital and temporal), and (2) two subtypes of long-distance PLIs (a) five interhemispheric PLIs (PLI scores between the left and right frontal, central, parietal, temporal and occipital MEG region) and (b) 8 (four per hemisphere) intrahemispherical PLIs (PLI scores between two different MEG regions, respectively left and right fronto-temporal, fronto-parietal, parieto-occipital and occipito-temporal).

### Graph analysis

The first step in the computation of the clustering coefficient (C) and path length (L) was to convert the 149 × 149 synchronization matrix into a binary (unweighted) graph by using a threshold, which means that PLI values above the threshold indicate existing edges. This binary graph is a network consisting of elements or vertices corresponding to MEG channels. Connections between these vertices are called edges. The clustering coefficient of a vertex (which is a measure of the local structure) can be computed by first determining to which neighboring vertices it is directly connected. The clustering coefficient is the ratio of all existing edges between the neighbors and the maximum number of edges possible between these neighbors, ranging between 0 and 1. This clustering coefficient is computed for all the vertices and averaged. The path length (indicating how well the network is integrated) is the average shortest path connecting any two vertices of the graph. The length of a path is indicated by the number of edges it contains. As we expected the structure of the graph to be influenced by the absolute number of edges per vertex of the graph, we computed the C and L as a function of degree *K*, meaning that the computed graphs have a fixed average number of edges per vertex. We choose *K *= 10 corresponding to our previous manuscript [8], although the choice of *K *is rather arbitrary.

To normalize variables, we computed the average C and L of 50 surrogate random graphs with the same degree and degree distribution as the epochs analyzed. This made it possible to compute the ratios between the C and L for each subject and the average of 50 random networks C/<C-s> and L/<L-s> (<> denoting ensemble averages).

Apart from these ratios, we computed the network 'small world-ness' S, based on the trade off between high local clustering and short path length. The small world-ness S is defined as the ratio (C/<C-s>)/(L/<L-s>) [38]. A network can be defined as a small-world network if C/<C-s> >> 1 and L/<L-s> ~ 1, which means that a value of S greater than 1 is called a small-world network.

We also computed the degree correlation (R) which indicates whether the degree of a vertex is influenced by the degree of another vertex to which it connects. The degree correlation can have a positive or negative value. When graphs have a positive degree correlation this indicates that vertices with high degrees are preferably connected to other vertices with high degrees.

### Statistical analysis

Differences between both groups in the distribution of age, sex, and education were analyzed by means of chi-square tests. Mann-Whitney nonparametric *U*-tests were used to investigate whether patients' standardized *z*-scores on neurocognitive tests in the overall measure of cognition differed significantly from healthy control *z*-scores.

Regarding the risk for type 1 errors that could interfere with the results, we tried to overcome the effect of multiple comparisons by normalizing the PLI scores to allow parametric testing. We normalized PLI scores by means of a logarithmic transformation [39]. To quantify differences in the PLI scores between the patients and the controls, we used an ANCOVA with repeated measures for each frequency band. The repeated measure factor had 8 levels in case of the global connections, 5 levels in case of interhemispheric connections, and 10 focal connection levels. The between-subjects factor had two levels (LGG patients and controls) and age, sex and education were used as covariates. In case of a significant effect for group or an interaction effect involving group (Greenhouse-Geisser corrected *p*-value), subsequent post hoc regression analyses with regard to the regional differences in PLI were done between the patients and the controls. Again age, sex and education were used as covariates.

For the graph analysis we calculated C/<C-s> and L/<L-s>, small world-ness (S), and degree correlation (R). The calculated values within each of the seven frequency bands were normally distributed according to the Kolomogorov-Smirnov test, except for the L/<L-s> ratio in the theta and lower alpha band within the patient group. In the ANCOVA, age, sex, and education level were used as covariates to evaluate whether true differences were present between the patients and the control population.

All analyses of the relation between higher neurocognitive function and network characteristics within the patient population involved separate ANCOVAs with repeated measures for each frequency band. The repeated measure level had 4 levels for the network characteristics (C/<C-s>, L/<L-s>, S and R). The age, sex, education level, tumor lateralization, treatment modalities (radiotherapy, surgery), and epilepsy burden were used as covariates together with one of the neurocognitive domains. In case of a significant effect for group or an interaction effect involving group (Greenhouse-Geisser corrected *p*-value), subsequent post hoc regression analyses with regard to these network characteristics were done with one of the neurocognitive domains and the above mentioned possible confounders as covariates.

## Results

### Sociodemographic and clinical characteristics

Thirty LGG patients met the inclusion criteria and seven patients declined to participate because they thought it would be too burdensome. Therefore, twenty-three out of thirty LGG patients were included. We subsequently had to exclude another six patients due to metal artifacts on the MEG (4 patients), ongoing epilepsy (1 patient), and tumor progression at the time of MEG registration (1 patient). The metal artifacts in 4 patients were induced by dental implants or amalgam fillings that had become magnetized during previous MRIs. The final analyses were performed on a sample of 17 patients and 17 matched healthy control participants. There were no significant differences in age, sex, and educational level due to the matching procedure.

Fourteen of the 17 LGG patients had a histologically confirmed LGG at the time of the MEG registration, and were operated 1–19 years (mean 8 years) before. Two patients were operated during the analysis of the data, within one year after the MEG registration. Of the 16 patients with a histologically confirmed LGG, the pathological diagnosis was grade II astrocytoma in ten patients, grade II oligodendroglioma in four patients and grade II oligo-astrocytoma in another two patients. Seven of the 17 patients underwent radiotherapy before the MEG registration with prior chemotherapy in two patients (1 patient with 5 cycles of PCV and 1 patient with 2 cycles of PCV and 3 cycles of temozolomide).

The tumor was localized in the left hemisphere in 11 patients and localized in the right hemisphere in 6 patients. Furthermore, we indexed the epilepsy burden of the patients as described by Klein et al. [36]. This scale has 6 levels: (level 1) epilepsy-free; (level 2) epilepsy, seizure-free in the year before testing without AEDs; (level 3) epilepsy, seizure-free in the previous year with AED monotherapy; (level 4) epilepsy, seizure-free in the previous year with AED polytherapy; (level 5) epilepsy, less than six seizures in the previous year and on AED monotherapy or polytherapy; and (level 6) epilepsy, more than six seizures in the previous year and on AED monotherapy or polytherapy. In the patient group, all but one patient used AED mono- or polytherapy. Six of the 17 patients with AED were free of seizures and 10 patients were still having seizures while on AED mono- or polytherapy.

### Differences between patient and healthy control group

#### Neurocognitive functioning

As previously described, six of the 17 patients had received neurocognitive assessments 1 to 9 months earlier and were clinically stable [9,26]. These patients were not tested again and the data from this last assessment were used, as their neurological status had not changed. Overall, patients performed poorer than healthy controls on the neurocognitive test battery (controls *M *≈ 0.00): they had a significantly lower *z*-score on the overall measure of cognition (*M *= -1.01, *p *= 0.009) than did control participants.

Furthermore, LGG patients had significantly poorer psychomotor functioning (*M *= -0.50, *p *= 0.035), poorer working memory (*M *= -1.43, *p *= 0.003), slower information processing speed (*M *= -0.85, *p *= 0.011), and decreased attentional capacities (*M *= -1.92, *p *= 0.003) relative to healthy controls. Patients' performance on the other two domains (e.g., verbal memory and executive functioning) did not differ statistically significant from controls.

To determine whether the small sampled group was representative of LGG patients in general, mean neuropsychological test scores were compared to a much larger group of patients (*n *= 195) from a previous study of our group [2]. The mean neuropsychological test scores of our patient group did not deviate more than 1 SD from this large group participating in that study. The observed larger SD in our study is most likely to be caused by smaller sample size.

### Functional connectivity

**Figure 1:**
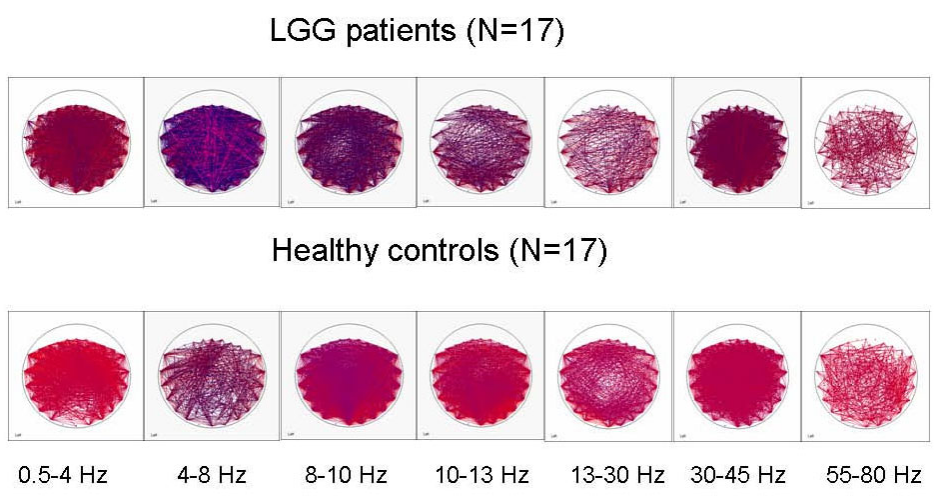
**A graphic representation of the differences in connectivity between the 17 LGG patients and the healthy control group for each frequency band**.

Figure 1 is a graphic representation of the differences in connectivity of the 17 LGG patients n and the healthy control group for each frequency band. A significant effect for group was only seen in the theta band for short-distance and interhemispheric connectivity (ANCOVA with repeated measures; respectively *p *= 0.009 and *p *= 0.046). Post hoc regression analysis showed that the intertemporal PLI was higher in LGG patients (M = 0.131, SD = 0.054) when compared to healthy controls (M = 0.104, SD = 0.018; F(1–32) 0.124; *p *= 0.044). The average PLI value in the right parietal region was also higher in LGG patients (M = 0.101, SD = 0.014) as compared to healthy controls (M = 0.087, SD = 0.012; F(2–32) 0.296; *p *= 0.005).

### Graph analysis

For the seven frequency bands, the value of the clustering coefficient (C/<C-s>) in both the patient and control group (mean respectively 2.05 and 2.08) was higher than in random networks, where C/<C-s> is expected to be around 1. The path length was slightly higher than that of random networks in both patients and controls (mean respectively 1.26 and 1.20).

**Figure 2:**
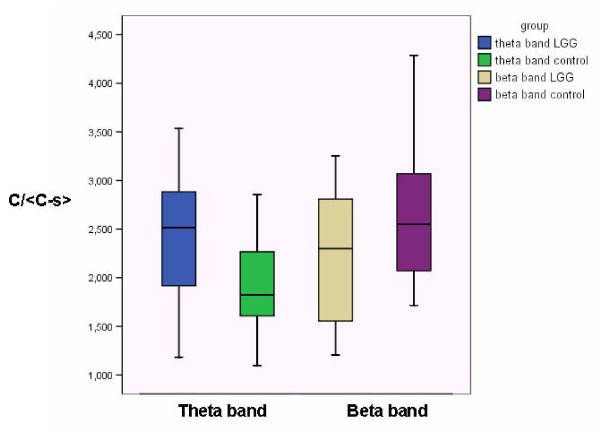
**Significant differences in the clustering coefficient in the theta and beta band between LGG patients and healthy controls**.

As shown in table 2, differences in network characteristics between patients and controls were found within the theta, beta, and upper gamma band. In the theta band a significantly higher C/<C-s> ratio was found in the patient population (ANCOVA; *p *= 0.021) compared to the healthy controls, whereas the opposite was true for the beta band (ANCOVA; *p *= 0.049) (Figure 2). Within the beta band, the patient population also showed a lower small world-ness S (ANCOVA; *p *= 0.030) compared to the healthy controls.

**Table 2:** Unweighted analysis with a fixed K at 10 of the differences between patients and controls concerning the network characteristics.

**Frequency band**	**Network characteristics**	**patient**	**control**	***p*-level**^a^
				

**Delta**	-			

				

**Theta**	C/<C-s>	**2.420 **(0.640)	1.886 (0.482)	0.021

				

**Lower alpha**	-			

				

**Upper alpha**	-			

				

**Beta**	C/<C-s>	2.199 (0.671)	**2.686 **(0.843)	0.049

	S	1.747 (0.499)	**2.122 **(0.455)	0.030

				

**Lower gamma**	-			

				

**Upper gamma**	R	-0.007 (0.063)	**0.054 **(0.066)	0.006

The higher value is depicted in bold and de standard deviation between parentheses.

Within each of the seven frequency bands, a negative degree correlation (R) was found in the patient population. In the upper gamma band, a significantly lower degree correlation was found in the patient population compared to the healthy controls (ANCOVA; *p *= 0.006).

### Association between network characteristics and neurocognitive functioning in the patient population

Within the seven frequency bands, significant differences were observed within the delta and lower alpha band. Significant effects for group in addition to an interaction effect was seen in the delta band between the factor network characteristics and executive functioning (ANCOVA with repeated measures; respectively *p *= 0.038 and *p *= 0.022), and in the delta band interactions were seen between network characteristics and executive functioning and attentional tasks (respectively *p *= 0.022 and *p *= 0.049). Post hoc regression analysis showed that an increase in the L/<L-s> ratio was associated with poorer executive and attentional functioning as shown in table 3 (*p *= 0.007 and *p *= 0.000 respectively). A higher degree correlation (R) was associated with better attentional functioning (*p *= 0.027).

**Table 3:** Significant associations in patients between network characteristics and neurocognitive function per frequency band and accompanying statistics.

						**model**		
**Frequency**	**Network characteristics**	**Neurocognitive domain**	**b**	**SEb**	**ß**	***R***^2^	**F**	***p***

								

**Delta (0.5–4 Hz)**	L/<L-s>	Executive functioning	-0.063	0.020	-0.624	0.389	1–16	0.007

	L/<L-s>	Attention	-0.040	0.008	-0.782	0.611	1–16	0.000

	R	Attention	0.016	0.007	0.533	0.284	1–16	0.027

								

**Lower alpha (8–10 Hz)**	C/<C-s>	Verbal memory	-0.230	0.100	-0.512	0.262	1–16	0.036

	L/<L-s>	Verbal memory	-0.262	0.084	-0.626	0.391	1–16	0.007

	R	Verbal memory	0.053	0.021	0.549	0.301	1–16	0.023

b = regression coefficient, SEb = standard error of b, *R*^2 ^= explained variance (L. = left, R. = right, inter = between hemispheres).

In the lower alpha band, a significant interaction effect was seen between the factor network characteristics and verbal memory (ANCOVA with repeated measures; *p *= 0.029). Post hoc regression analysis showed that increases in C/<C-s> and L/<L-s> were associated with decreasing verbal memory (*p *= 0.036 and *p *= 0.007 respectively). On the other hand, increasing degree correlation was associated with better verbal memory (*p *= 0.023).

## Discussion

In this study we show 1) higher synchronization in the theta frequency band and differences in neural network organization in LGG patients compared to healthy controls, and 2) associations of changes in overall network organization to be related with neurocognitive function.

By using the phase lag index (PLI), which is a novel method to detect synchronous neural activity of the MEG recordings, we showed differences in synchronization between LGG patients and healthy controls. Within the theta frequency band, short- and long-distance synchronization was significantly higher in LGG patients compared to healthy controls. Functional connectivity in brain tumor patients has been studied before by using the synchronization likelihood (SL) as a different measure of statistical interdependencies between two time series. In a study of Bartolomei et al., increased short- and long-distance synchronization in the delta, theta, and alpha frequency band were observed in patients with a variety of primary brain tumors [8]. The SL has also been computed in the present LGG population and increased long-distance synchronization was found in the delta, theta, and gamma frequency band, and a decline in the alpha frequency band was found within the patient population [9]. The current study again shows differences in functional connectivity between brain tumor patients and healthy controls in the theta band. The PLI is a conservative measure of functional connectivity, so the finding of pathologically increased theta band functional connectivity in LGG seems to be a robust finding. Previously observed differences in synchronization in the other frequency bands could have been a result of volume conduction, although underestimation of the true synchronization (with PLI) is possible since this measure excludes synchronization expressed in the near zero phase coupling range. Our current results therefore strongly support the hypothesis that brain tumors lead to significant changes in theta band connectivity.

There is growing evidence for pathologically increased synchronization in brain tumor patients within the lower frequency bands [8-10]. In one recent study of our group, the PLI was used to compare functional connectivity in brain tumor patients before and after resective surgery [10]. Remarkably, brain tumor patients in this study showed a decrease in theta band synchronization after surgery compared to their preoperative status. Moreover, those patients displaying a major decrease in synchronization were more often free of epilepsy after surgery compared to the patients with just a small decrease in synchronization These findings suggested that in fact preoperative theta band synchronization might have been abnormally high, as is confirmed in the present study. Increased synchronization in the theta frequency band has been observed before in other patient groups, such as Alzheimer's disease [40], autism spectrum disorders (ASD) [41], and major depression [42]. Furthermore, associations between theta band activity neurocognitive performance such as working memory processes and attentional functioning has been observed before [43,44].

By analyzing the spatial configuration of brain networks in the LGG patients compared to healthy controls we observed that for both patients and controls the clustering coefficient was twice as high as the value of the clustering in random networks whereas the path length was just slightly higher than in random networks. Therefore, brain networks show a small world configuration in both patients and controls. Within the theta band the clustering coefficient was significantly higher in the patient population compared to the controls, whereas the opposite was true for the beta band (e.g. clustering was lower in the patient population). The path length remained fairly stable over all frequency bands compared to healthy controls. Local clustering is increased in LGG patients in the lower frequency band (more small world organization) and decreased (more random) in the higher frequency bands. The differences in the beta band with respect to clustering were also reflected in the lower value of S (as a measure of small world-ness) in LGG patients compared to healthy controls.

Bartolomei et al. previously reported the neural architectural properties in a group of patients with various brain tumors by using graph analysis and compared these results to a healthy control population [8]. That study showed lower clustering coefficients and shorter path length in the brain tumor population in the theta and gamma band. In the beta band, brain tumor patients only displayed shorter path lengths with relative sparing of the clustering coefficient. In the present study, we also observed changes in the theta and beta band, although they were more prominent at the level of local clustering. Apart from that, the present study showed an increase in local clustering in LGG patients in the lower frequency band whereas Bartolomei showed a decrease. Several methodological differences might have contributed to the observed differences. In the previous study, patients with a mixture of primary brain tumors were evaluated whereas we only included LGG patients, also resulting in differences in tumor treatment between the two groups. Secondly, our healthy control population was age- sex-, and education-matched whereas the control population of Bartolomei was significantly younger than the patient group. The influence of ageing on network structures has been described previously [19]. Thirdly, Bartolomei used the SL to apply graph analysis whereas we used the PLI. Since differences between both measures were found with respect to analysis of coupling, differences in the outcome of graph analysis can be expected. We observed that the clustering ratio (C/<C-s>) was twice as high in the study of Bartolomei compared to the patients in the current study, but since path length remains rather short, both patient populations still display a small world configuration. Although the two brain tumor patient groups differed at the level of patient and tumor characteristics and the used method of analysis, both groups do show changes in the overall organization of neural networks compared to healthy controls.

Our study is the first to correlate neurocognitive function to graph theoretical parameters in brain tumor patients. Increased path length was associated with poorer executive functioning and attentional task performance in the delta band, and was associated with decreasing verbal memory within the lower alpha band. Increased local clustering was also associated with poorer verbal memory in the lower alpha band. Within both the delta and lower alpha band, a lower degree correlation was associated with diminished attentional functioning and verbal memory. Interpreting these results remains rather speculative. It is possible that patients with a longer path length due to disconnection show more neurocognitive deficits. Local clustering in the lower frequency bands was significantly higher in LGG patients compared to healthy controls, which could be interpreted as a compensatory mechanism. Micheloyannis et al. [18] found differences in the network organization between subjects with a high and low education during a working memory task. They observed a less prominent small-world organization in the high education subject group compared to the more prominent small world network organization in the low educated subject group, suggesting that those with a lower cognitive ability need to optimize their neuronal organization to perform well during working memory tasks, whereas this is not the case in the high educated group.

Secondly, a lower degree correlation was associated with decreased neurocognitive performance. It can be expected that for optimal organization of a network (e.g. optimal neurocognitive performance), vertices with higher degrees are preferably interconnected. A randomly organized network does not show this preferred coupling and therefore will have a degree correlation ~0. In our population, we observed a negative value for the degree correlation within every frequency band which can be caused by the fact that we used the PLI, which can be rather insensitive to the detection of true connectivity between sensors at a short distance.

In other patient groups the graph theoretical properties have been studied before at both resting state and during cognitive tasks. In a study of Stam et al., Alzheimer disease patients (AD) were compared with patients with only subjective memory complaints, and a loss of small-world features was found in the beta band. The authors showed that these AD patients had a longer path length with relative sparing of local clustering [22]. In this study a higher score on the Mini Mental State Examination (MMSE) was correlated with shorter path length. Remarkably, we also observed that an increased path length was associated with poorer neurocognitive functioning. In another previous study of Stam et al [23] PLI-weighted connectivity networks were calculated and characterized in Alzheimer disease patients and non-demented controls showing a decreased clustering coefficient and shorter path length (closer to a random network) in the Alzheimer disease patients within the lower alpha band. No significant correlation was found between the MMSE and PLI or network measures within the Alzheimer patient group. Smit et al. evaluated the clustering coefficient and average path length in monozygotic and dizygotic twins and their singleton siblings by using resting state EEG registrations. They performed graph analysis to see whether there was a genetic attribution to the observed results, and concluded that the clustering coefficient, path length and the small-world-ness measure (S) are viable markers of genetic differences in brain organization. Additionally, they correlated graph parameters with four neurocognitive domains (verbal memory, working memory, psychomotor speed, and perceptual organization) and found no correlation between neurocognitive function and organization of neural networks [20]. In schizophrenia patients, subtle randomization of the network architecture was found compared to healthy controls [25]. Furthermore, healthy controls showed a high local clustering and a relative short path length in the alpha, beta and gamma band during a working memory task, whereas a loss of small-world properties was found in the schizophrenic patients [24].

From our study we can conclude that the disturbed organization of a complex network structure in glioma patients seems to be associated with neurocognitive dysfunction. Although our patient population consisted of a homogeneous group of brain tumor patients, the influence of tumor treatment on the network organization and the influence on neurocognitive function cannot be answered with this cross-sectional study. Further research is needed, and a longitudinal study is currently under way in our department. On the basis of the results of the present study, we will evaluate the strength and spatial organization in brain tumor patients before and after treatment, and correlate these results with higher neurocognitive function.

## Competing interests

The authors declare that they have no competing interests.

## Authors' contributions

JCR participated in the design of the study and the manuscript, MK participated in the design of the study and the manuscript and supported the neuropsychological assessments of the study subjects, LD conceived of the study and supported with the statistical analysis, BWvD participated in the design of the study and supported with the MEG recordings and the analysis of the data, JJH participated in the design of the study and the manuscript and CJS participated in the design of the study and manuscript and supported with the analysis of the data. All authors read and approved the final manuscript
